# Function and mechanism of toll-like receptors in cerebral ischemic tolerance: from preconditioning to treatment

**DOI:** 10.1186/s12974-015-0301-0

**Published:** 2015-04-28

**Authors:** Peng-Fei Wang, Xiao-Yi Xiong, Jing Chen, Yan-Chun Wang, Wei Duan, Qing-Wu Yang

**Affiliations:** Department of Neurology, Xinqiao Hospital & the Second Affiliated Hospital, the Third Military Medical University, No. 183, Xinqiao Main Street, Shapingba District Chongqing, 400037 China; Department of Neurology, Weihai municipal Hospital, Weihai, 264200 China

**Keywords:** Preconditioning, Postconditioning, Ischemic tolerance, Inflammation, Toll-like receptors, TLRs signaling

## Abstract

Increasing evidence suggests that toll-like receptors (TLRs) play an important role in cerebral ischemia-reperfusion injury. The endogenous ligands released from ischemic neurons activate the TLR signaling pathway, resulting in the production of a large number of inflammatory cytokines, thereby causing secondary inflammation damage following cerebral ischemia. However, the preconditioning for minor cerebral ischemia or the preconditioning with TLR ligands can reduce cerebral ischemic injury by regulating the TLR signaling pathway following ischemia in brain tissue (mainly, the inhibition of the TLR4/NF-κB signaling pathway and the enhancement of the interferon regulatory factor-dependent signaling), resulting in TLR ischemic tolerance. Additionally, recent studies found that postconditioning with TLR ligands after cerebral ischemia can also reduce ischemic damage through the regulation of the TLR signaling pathway, showing a significant therapeutic effect against cerebral ischemia. These studies suggest that the ischemic tolerance mediated by TLRs can serve as an important target for the prevention and treatment of cerebral ischemia. On the basis of describing the function and mechanism of TLRs in mediating cerebral ischemic damage, this review focuses on the mechanisms of cerebral ischemic tolerance induced by the preconditioning and postconditioning of TLRs and discusses the clinical application of TLRs for ischemic tolerance.

## Introduction

Ischemic stroke, a current major disabling and killer disease worldwide, is characterized by a high incidence, morbidity, and disability rate. However, to date, only r-tPA intravenous thrombolysis has been proven to be an effective therapy [1]; other effective treatments are still lacking. However, the narrow time window (3 to 4.5 h after onset) and the reperfusion damage after thrombolysis in r-tPA intravenous thrombolytic therapy severely limit its clinical application. Therefore, studies of cerebral ischemic injury in recent years have focused on revealing and inducing the endogenous protective mechanism of cerebral ischemic injury, improving the protective effect against cerebral ischemia, and alleviating cerebral ischemic injury. As far back as the 16th century, the toxicologist Paracelsus observed that ‘the dose makes the poison’. A corollary is that subtoxic doses of cellular stress can lead to the generation of a protective state, termed ‘preconditioning’ [2]. Preconditioning is a procedure by which a noxious stimulus near to but below the threshold of damage is applied to the tissue. Shortly after preconditioning, or after a delay, the organ develops resistance to, or tolerance of, the same stimulus or even different noxious stimuli given beyond the threshold of damage. Preconditioning thereby protects against subsequent injury [3]. Such endogenous modulators mediate the phenomenon of ischemic tolerance, which was first identified in the brain in the early 1990s and has been shown to have a neuroprotective role in cerebral ischemia [4]. In recent years, increasing evidence has suggested that toll-like receptors (TLRs) mediate inflammatory cerebral ischemic injury and play an important role in the secondary brain injury following cerebral ischemia [5-7]. Additionally, preconditioning with TLR ligands reduces cerebral ischemic damage, leading to ischemic tolerance. Importantly, recent studies have found that the postconditioning of TLRs also reduces cerebral ischemia reperfusion damage; therefore, TLR-mediated ischemic tolerance has an important transforming significance in the prevention and therapeutic targets of cerebral ischemic injury. On the basis of describing the function and mechanism of TLRs in mediating cerebral ischemic damage, this review focuses on the mechanisms of cerebral ischemic tolerance induced by TLR preconditioning and postconditioning and discusses the clinical application of TLR ischemic tolerance.

### Introduction of TLRs and their signaling pathways

TLRs are a group of classic pattern recognition receptors (PRRs) that can recognize the pathogen-associated molecular patterns (PAMPs) from pathogenic microorganisms and injury-related damage-associated molecular patterns (DAMPs) [8]. To date, 11 TLRs have been found in humans, and 13 TLRs have been identified in mice; TLR1 to TLR9 are common to both species [9]. TLR2 can recognize lipopeptides; it may form a heterodimer with TLR1 to recognize trimerized lipopeptides and with TLR6 to recognize dimerized lipopeptides [9]. TLR3 recognizes double-stranded viral RNA and polycytidylic acid (poly-IC) and mediates the activation of NF-κB and the type I interferon (IFN) signaling pathway [10,11]. TLR4 recognizes lipopolysaccharide (LPS) as a cell wall component of gram-negative bacteria [12]; TLR5 recognizes the flagellin component of bacterial flagella and regulates the natural and acquired immunity of intestinal bacteria [13-15]. TLR7 and its related TLRs sense single-stranded viral RNA [16-18], and TLR9 recognizes the CpG-DNA pattern and reacts with herpes viral DNA [19-21]. TLR11 and TLR12 in mice associate to detect bacterial components [8,22,23]. Furthermore, TLRs can also recognize the endogenous molecules released by damaged cells, suggesting that the innate immune inflammatory reaction can be activated by the endogenous ligands released from damaged tissue in the absence of foreign pathogens.

On the basis of a specific adapter, a TLR signal can be conducted through a MyD88-dependent or MyD88-independent signal. In general, except for TLR3, the signals of all TLR family members are initiated by the MyD88 protein and conducted through the MyD88-dependent signaling pathway. TLR2 and TLR4 can bind to MyD88 after successfully assembling with TIRAP/Mal, and after binding to IL-1R-associated kinase (IRAK)-4, MyD88 can activate other members of the IRAK family, such as IRAK-1 and IRAK-2 [24]. Then, IRAKs and MyD88 disassociate and react with the TNFR-associated factor 6 (TRAF6). Together with an E2 ubiquitin-conjugating enzyme complex comprising Ubc13 and Uev1A, TRAF6 catalyzes the formation of a lysine 63 (K63)-linked polyubiquitin chain on TRAF6 itself as well as the generation of an unconjugated free polyubiquitin chain [25]. A complex of TGF-b-activated kinase 1 (TAK1), TAK1-binding protein 1 (TAB1), TAB2, and TAB3 is activated by MAP kinase 6. Subsequently, IKK-a, IKK-b, and NF-κB form an IKK complex to phosphorylate IκB, thereby freeing NF-κB to translocate into the nucleus and activate the expression of the pro-inflammatory cytokine genes. The activation of the MAP kinase cascade is responsible for the formation of another transcription factor complex, AP-1, which targets cytokine genes [26].

TLR3 recognizes dsRNA and binds to TIR-domain-containing adapter-inducing interferon-β (TRIF). TLR4 triggers both MyD88- and TRIF-dependent signaling pathways. After the binding to TLR4 and TRAM, TRIF is activated. TRIF associates with TRAF3 and TRAF6 through the TRAF-binding motifs present in its N-terminal portion [26]. TRAF3 is the key factor to activate TANK-binding kinase 1 (TBK1) and IKK-i [27,28]. TBK1 and IKK-α phosphorylate interferon regulatory factor (IRF)3 and IRF7; when the dimer of IRF3 and IRF7 is translocated into the nucleus, the induction of type I IFNs and the expression of IFN-inducible genes occur. Additionally, TRAF6 and RIP1 activate NF-κB, which induces the production of the pro-inflammatory cytokines [26].

TLR7 and TLR9 signaling induces the production of type I IFNs in a MyD88-dependent manner, in addition to NF-κB-dependent cytokines. MyD88 forms a complex with IRAK-1, TRAF6, TRAF3, IKK-α, and IRF7, and phosphorylated IRF7 translocates into the nucleus to activate the expression of genes encoding type I IFNs [26].

### Toll-like receptors and cerebral ischemic injury

Post-ischemic inflammation is an important pathophysiological process in the development of ischemic stroke, but excessive inflammation can promote neuronal apoptosis and aggravate neurological impairment [29]. TLRs mediate the inflammatory response in immune cells, suggesting that they also participate in mediating the inflammation and the secondary brain injury following ischemia. It is now clear that TLRs play an important role in cerebral ischemia [30]. Injured neurons release endogenous DAMPs to activate TLRs and further stimulate inflammatory cascades to induce secondary injury [31]. However, the complex mechanism of TLR-mediated neuronal injury and the exact role of TLRs have not yet been fully clarified.

#### TLR2 and cerebral ischemic injury

Recent studies have shown that the expression of TLR2 mRNA in focal ischemic brain tissue was significantly increased, and TLR2 was the most strongly upregulated TLR [5,32]. Additionally, compared with wild-type mice, the area of infarction in TLR2-deficient mice was significantly reduced [32]. Moreover, in the ischemic brain tissue, the common signaling pathway mediated by the TLR2/1 dimer and CD36 induced ischemic inflammation and tissue damage [33]. These results indicate that TLR2 plays an important role in the inflammatory injury that follows cerebral ischemia.

Recent studies have shown that, compared with a control group, the number of CD11b-positive cells was significantly reduced, and there was a significant reduction in the neuronal death rate in C57BL mice arterially injected with a TLR2 antibody (clone T 2.5) 45 min after cerebral ischemia [34]. In clinical observations, TLR2 expression in the peripheral blood cells of patients with ischemic stroke was significantly upregulated. The addition of the peripheral blood from patients with stroke into mononuclear cells and human umbilical cord vein endothelial cells induced a significant inflammatory reaction, and this reaction could be inhibited by a TLR2 antibody [6]. These results suggest that blocking the TLR2 signal can alleviate cerebral ischemic inflammation, and TLR2 is expected to serve as an important intervention target for the treatment of cerebral ischemia.

However, one report showed that 24 h after cerebral ischemia-reperfusion injury, the area of cerebral infarction in TLR2-knockout mice was increased. This study indicated that TLR2 knockout did not inhibit NF-κB activation; on the contrary, it inhibited the activation of the PI3K/Akt signaling pathway, thereby aggravating the cerebral injury caused by ischemia reperfusion [35]. The exact reason for the difference between the above observed results is not yet clear, but it may be associated with differences in animal models, suture size, body weight, ischemia-reperfusion time, and cerebral blood flow.

Recent studies have shown that, compared with wild-type mice, TLR2 knockout reduced the proliferative capacity of the microglia in ischemic brain tissue, reduced the MCP-1 level, and decreased the accumulation of the CD45^+^/CD11b^+^ cells at the site of cerebral ischemic injury, leading to a worsened delay of cerebral ischemic injury, which manifested as an expanded area of chronic cerebral infarction [36]. These results further suggest that the function of TLR2 in cerebral ischemia-reperfusion injury awaits additional investigation, especially for its effect on the cerebral infarction in the chronic stage.

TLR2 mRNA and proteins are expressed in the nerve primitive cells (NPCs) in the embryo, neonatal mouse, and adult mouse as well as in *in vitro* NPC cultures [37,38]. The TLR2 ligand Pam3CSK4 or an endogenous ligand of low molecular weight hyaluronic acid inhibits the formation of neurospheres *in vivo*, showing that TLR2 activation inhibits the proliferation of embryonic NPCs [38]. In adult mice, a TLR2 defect impairs the regeneration of hippocampal neurons, but TLR2 activation did not damage NPC proliferation in adult mice [39]. These results suggest that TLR2 is involved in nerve regeneration and remodeling, but the effect of TLR2 on the nerve regeneration and tissue repair with cerebral ischemic injury requires further investigation.

#### TLR3, TLR9, and cerebral ischemic injury

After 2 h of middle cerebral artery occlusion (MCAO) followed by reperfusion for 22 h, the volume of cerebral infarction in TLR3- or TLR9-deficient mice was not significantly reduced, and there was no improvement in the neurological score [40], suggesting that TLR3 or TLR9 had no protective effect on MCAO-induced ischemic cerebral damage. Additionally, clinical studies have also shown that the expression of TLR3 or TLR9 in the peripheral blood of patients with ischemic stroke did not correlate with the neurologic impairment [41], which further suggests that TLR3 and TLR9 are not directly involved in the regulation of inflammatory injury of cerebral ischemic reperfusion (I/R). However, it has been demonstrated that TLR3 signaling can activate the NF-κB to produce pro-inflammatory cytokines, and the TLR3-deficient mice showed reduced production of inflammatory cytokines [42]. Moreover, recent studies have also shown that TLR3 signaling inhibits hippocampal neurogenesis [43-45], which has been ascribed to inflammation in the adult brain [46]. These results suggest that TLR3 signaling is harmful to brain injury.

#### TLR4 and cerebral ischemic injury

TLR4 plays an important role in innate immunity and the cerebral ischemic injury of the central nervous system. Our previous studies showed that the infarction area and the neurological impairment of the TLR4-knockout mice in focal MCAO were significantly reduced [47]. Subsequently, the whole cerebral ischemia model further confirmed that the cerebral ischemic damage in TLR4-knockout mice was also significantly reduced [7]. Similarly, the degeneration of retinal ganglion cells (RGCs) induced by ischemia and axotomy could be reduced in TLR4-knockout mice, and the phosphorylation levels of ERK-1/-2, c-Jun N-terminal kinase (JNK)-1/-1, and p38 were significantly decreased, with a low level of inducible NO synthase [48]. *In vitro* experiments showed that after oxygen-glucose deprivation (ODG), the survival rate of cultured cortical neurons from TLR4-knockout mice was significantly increased [49]. Additionally, spinal cord I/R injury also led to TLR4 upregulation and microglial activation, which initiates neuroinflammation and neuroapoptosis via the NF-κB/IL-1β pathway [50]. Clinical studies have further confirmed that the TLR4 expression in neutrophils was associated independently with patients’ prognoses [51]. These results suggest that TLR4 mediated the post-ischemic inflammation, and the inhibition of TLR4 activation could significantly reduce cell damage *in vivo* and *in vitro*.

Our previous results showed that the number of TLR4^+^ monocytes in the peripheral blood of patients with cerebral infarction was significantly increased, and the correlation analysis showed that TLR4 expression and cytokine levels were closely related to stroke severity [52]. Additionally, after adding the serum of patients with cerebral ischemia into the cultured monocytes and human umbilical vein endothelial cells, a strong inflammatory response occurred, which was blocked by the addition of a TLR4 antibody [6]. Moreover, inhibition of the TLR4/NF-κB pathway decreased I/R brain and spinal cord injuries [50,53]. These above results further suggest that TLR4 is involved in the cerebral ischemic damage, suggesting that TLR4 is expected to become an important intervention target for cerebral ischemic damage.

The TLR signaling pathway has been shown to be involved in the inflammatory response following cerebral I/R, but the accompanying downstream TLR signaling events remain poorly understood. Interestingly, the infarct sizes were not decreased in *MyD88*- or *TRIF*-mutant mice compared to that in wild-type (WT) mice following focal ischemia [54]. A recent study showed that a compensatory Th2-type skew at baseline in *MyD88*^*−/−*^ mice and a paradoxical switch to a Th1 phenotype following focal cerebral ischemia; additionally, the MyD88 pathway directs the expression of neutrophil chemoattractants following cerebral ischemia [55]. In contrast, the MyD88-independent pathway and the ubiquitin-mediated proteolysis pathway may be the most vital molecules among TLR downstream pathways in incidences of ischemic stroke [56]. Therefore, the accompanying downstream TLR signaling following ischemia needs to be further explored.

Studies have shown that TLR4 plays an important role in NPC proliferation and differentiation. TLR4 is widely expressed in the NPCs of embryos, neonatal mice, and adult mice [39]; TLR4 defects can promote NPC proliferation and differentiation [39]. Additionally, a TLR4 inhibitor increases NPC proliferation and differentiation [39]. This result suggests that TLR4 signaling can negatively regulate NPC proliferation and differentiation in adult mice. However, a recent study showed that the area of skin wounds in *TLR4*^*-/-*^ mice was significantly larger; meanwhile, the expression of TGF-b and CCL5 was reduced, indicating that TLR4 can regulate wound healing by increasing TGF-b and CCL5 [57]. However, in the context of cerebral ischemia, the effect of TLR4 activation by endogenous ligands on the regeneration of nerve cells and tissue repair awaits further study.

#### TLR8 and cerebral ischemic injury

TLR8 is mainly expressed in cortical neurons. Studies have shown that TLR8 expression was significantly increased at 6 h after cerebral ischemia in mice, and in *in vitro* oxygen-glucose deprivation (OGD) in cells, the expression levels of TLR8 and the downstream JNK signaling pathway were also significantly upregulated [58]. However, after the specific inhibition of TLR8 using siRNA, the SH-SY5Y cells with OGD were significantly protected. Additionally, TLR8 activation enhanced the apoptotic and the inflammatory response after stroke mediated by T cells [58]. The above results show that TLR8 activation aggravated ischemic brain injury. Additionally, clinical studies have shown that TLR8 expression in the peripheral blood of patients with ischemic stroke significantly correlated with the prognosis of the patients [41], which further demonstrates that TLR8 is directly involved in the inflammatory damage of cerebral ischemia and hypoxia.

### Preconditioning with TLR ligands and cerebral ischemic injury

Many studies support the idea that innate immunity has a toxic effect in cerebral ischemic injury. As mentioned previously, TLR2 and TLR4 knockout reduces inflammation, decreases the cerebral infarction area, and improves the neurological function score in MCAO mice; therefore, targeting the TLR signal may become an important treatment strategy for cerebral ischemic injury [59]. Recent studies have found that a small dose of TLR ligand before cerebral ischemia generated a tolerance for the subsequent cerebral ischemic damage. For example, the application of LPS (a powerful TLR4 agonist) as preconditioning prior to cerebral ischemia reduced the subsequent cerebral ischemic injury and improved the neurological function score [60], which was termed LPS tolerance [61]. To date, it has been reported that preconditioning with TLR2, 3, 4, 7, and 9 ligands induces TLRs to generate ischemic tolerance and also reduces cerebral ischemic damage [62-66] (Table 1).

**Table 1:** **TLRs preconditionings and effects**

**Animal**	**Model**	**Preconditioning time/approach**	**TLR/ischemia**	**Result and mechanism**	**Author**	**Reference**
Mice	MCAO, 60-min ischemia, 24-h reperfusion	72 h/12-min ischemia	Ischemic preconditioning	Decrease brain infarction volume, and IRF gene participate in brain protection	Stevens SL	[79]
Mice	MCAO, 60-min ischemia, 6-h reperfusion	24 h/IP	TLR2, Pam3CSK4	Decrease brain infarction and edema, improve neurological function, and maintain BBB function	Hua F	[60]
Mice	MCAO, 45-min ischemia, 24-h reperfusion	72 h/subcutaneous injection	TLR3, poly-ICLC	Decrease brain infarction volume and improve neurological function	Packard AEB	[63]
Mice	MCAO, 120-min ischemia, 22-h reperfusion	24 h/systemic injection	TLR3 poly-IC	Decrease brain infarction volume and neurological defect and lower the levels of TNF-α and IL-6 in cortex and striatum	Pan LN	[66]
Mice	MCAO, 60-min ischemia, 24-h reperfusion	1 h/IP	TLR3 poly-IC	Decrease brain infarction and prevention of Fas/FADD interaction	Zhang X	[67]
Spontaneously hypertension rats	MCAO, 120-min ischemia, 22-h reperfusion	2, 3, 4 days/IV	TLR4 LPS	Decrease brain infarction volume, mediated by TNF-α	Tasaki K	[72]
Mice	MCAO, 120-min ischemia, 47-h reperfusion	48 h/IP	TLR4 LPS	Decrease brain infarction volume, inhibit the infiltration of neutrophil and activation of the microglia	Rosenzweig HL	[73]
Mice	MCAO, 40-, 45-, 60-min ischemia, 24-h reperfusion	72 h/IP	TLR4 LPS	Decrease brain infarction volume that was mediated by IRF3	Marsh B	[61]
Mice	MCAO, 40-to 60-min ischemia, 24-h reperfusion	72 h/subcutaneous injection	TLR4 LPS	Decrease brain infarction volume, and TRIF-IRF3 play vital role	Vartanian KB	[58]
Mice	MCAO, 60-min ischemia, 24-h reperfusion	72 h/IP	TLR4	Decrease brain infarction volume, and inhibit TNF-α signaling pathway after cerebral ischemia	Rosenzweig HL	[76]
Mice	MCAO, 45-to 60-min ischemia, 23-h reperfusion	72 h/subcutaneous injection	TLR7 GDQ	Decrease brain infarction volume and neurological functional score that was protected through I type interferon	Leung PY	[62]
Mice	MCAO, 60-min ischemia, 72-h reperfusion	30 min/IV	TLR8 R848	Increase neurological function defect and mortality, activate pro-apoptotic JNK pathway and inflammation	Tang SC	[56]
Mice	MCAO, 60-min ischemia, 24-h reperfusion	72 h/IP	TLR9 CpG-ODN	Decrease brain infarction volume and TNF-α play a vital role	Stevens SL	[64]
Mice	MCAO, 60-min ischemia, 24-h reperfusion	72 h/IP or subcutaneous injection	TLR9 CpG-ODN	Decrease brain infarction volume and IRF gene participate in cerebral protection after ischemia	Stevens SL	[79]
Mice	MCAO, 60-min ischemia, 24-h reperfusion	1 h/IP	TLR9 CpG-ODN	Decrease brain infarction and activation of PI3K/Akt signaling	Lu C	[78]

MCAO: middle cerebral artery occlusion; IP: intraperitoneal injection; IV: intravenous injection; HI: hypoxic-ischemic; ICA: internal carotid artery; IRF: interferon regulatory factor; TLR: toll-like receptor; BBB: blood-brain barrier; Poly-IC: polycytidylic acid; Poly-ICLC: polyinosinic-polycytidylic acid; FADD: Fas-Associated protein with Death Domain; LPS: lipopolysaccharide; IRF: interferon regulatory factor; TRIF: TIR-domain-containing adapter-inducing interferon-β; GDQ: gardiquimod; JNK: c-Jun N-terminal kinase; CpG-ODN: CpG-motif oligodeoxynucleotide. MCAO, 60-min ischemia, 24-h reperfusion: the mice was employed MCAO (cerebral ischemia) 60 min followed by reperfusion 24 h. 1 h/IP: mice were injected intraperitoneally with TLRs ligand 1 h prior to cerebral ischemia.

#### TLR2 ligand preconditioning and ischemic injury

Studies have shown that TLR2 induces ischemic tolerance [62]. Systematic injection of the TLR2 ligand Pam3CSK4 24 h before the focal MCAO cerebral ischemia, the significantly reduced volume of cerebral infarction in the Pam3CSK4-preconditioning mice were observed, as well as reduced brain edema, maintained nerve function, and reduced mortality in the acute phase [62]. It is important to note that Pam3CSK4 preconditioning can reduce the amount of serum globulin and maintain the function of the blood-brain barrier after cerebral I/R injury [62]. These results show that maintaining the function of the blood-brain barrier may be one of the mechanisms of Pam3CSK4 preconditioning in protecting cerebral ischemia.

**Figure 1:**
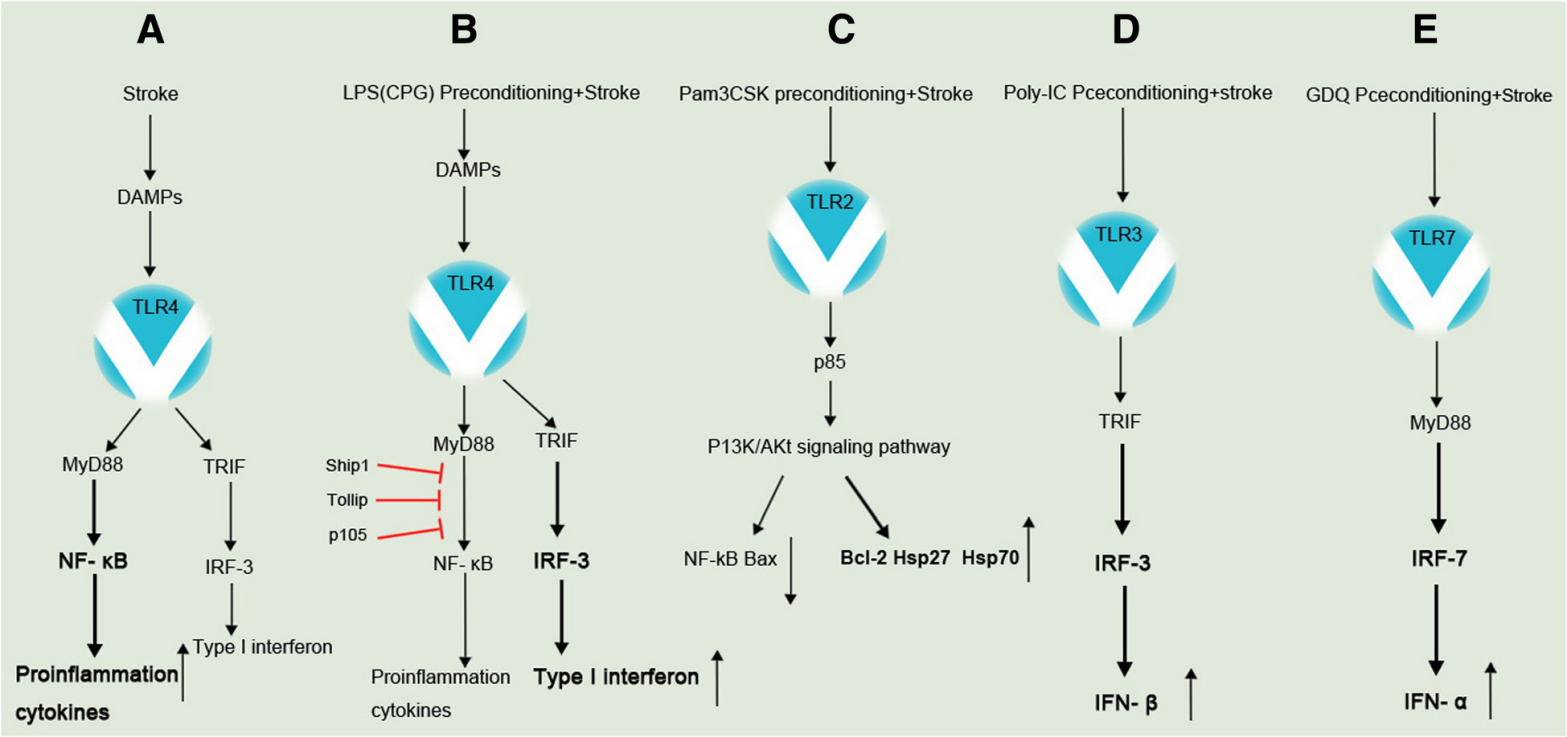
Schematic of TLR signaling and gene expression following stroke. **(A)** TLR4 signaling cascades following stroke. Stroke leads to NF-κB activation without IRF3 activation. **(B)** LPS (CpG) preconditioning prior to stroke leads to robust activation of IRF3 and type I interferon; meanwhile, the increased Ship1, Tollip, and p105 lead to the suppression of NF-κB activity and pro-inflammation cytokines compared to stroke alone. **(C)** Pam3CSK4 preconditioning activates the TLR2/PI3K/Akt signaling pathway and subsequently downregulates NF-κB activity and the expression of Bax, as well as increases the expression of Bcl-2, Hsp27, and Hsp70. **(D)** Poly-IC preconditioning activates IRF3 and induces IFN-β production. **(E)** GDQ preconditioning activates IRF7 and induces IFN-α production. DAMPs, damage-associated molecular patterns; TLR, toll-like receptor; TRIF, TIR-domain-containing adapter-inducing interferon-β; IRF, interferon regulatory factor; LPS, lipopolysaccharide; Poly-IC, polycytidylic acid; .

Additionally, the systematic injection of Pam3CSK4 1 h before the focal cerebral ischemia could also significantly reduce cerebral ischemic-reperfusion injury [67]. Moreover, NF-κB activity and Bax expression were significantly reduced, whereas the expression levels of Bcl-2, Hsp27, and Hsp70 were significantly increased. Furthermore, the protective effect of Pam3CSK4 preconditioning was implemented mainly through the TLR2/PI3K/Akt-dependent signaling pathway [67]. These results suggest that the Pam3CSK4 preconditioning for cerebral ischemic tolerance not only inhibits the inflammatory response but also plays a role in the anti-apoptotic mechanism and the brain protection of Hsp (Figure 1C).

#### TLR3 ligand preconditioning and cerebral ischemic injury

TLR3 ligand poly-IC preconditioning has also been demonstrated to induce ischemic tolerance [65]. The subcutaneous injection of poly-IC to mice 3 days before MCAO significantly reduced the infarction area, and this effect was dose dependent; importantly, despite the fact that the protective effect of poly-IC preconditioning was similar to the LPS preconditioning, poly-IC showed no significant toxicity [65]. Additionally, the systematic injection of poly-IC to mice 2 h before MCAO also reduced the cerebral infarction area and neurological impairment [68]. Our study found that the intraperitoneal injection of poly-IC 1 h before ischemia also had a protective effect for cerebral ischemia (unpublished data). These decreased cerebral I/R injury by poly-IC treatment may be via the TLR3-mediated prevention of Fas/Fas-Associated protein with Death Domain (FADD) interaction [69]. These results suggest that poly-IC preconditioning can significantly reduce the focal cerebral I/R injury. Poly-IC preconditioning also alleviated the death of mixed cortical cells after OGD [65] and alleviated OGD-induced astrocyte damage [68], indicating that poly-IC preconditioning can reduce the ischemic cell damage *in vitro* and has a protective effect on neurons and glial cells.

Poly-IC preconditioning can induce IFN-β expression in astrocytes and microglial cells of ischemic brain tissue [70] and maintain the bypass transporter function of endothelial cells to reduce the loss of transendothelial electronic resistance [70]. Moreover, type I IFN signaling in brain microvascular endothelial cells was required for the cerebral ischemic protection, suggesting that the protection of poly-IC preconditioning against cerebral ischemia was achieved mainly by the type I IFN signaling to maintain the blood-brain barrier. Additionally, poly-IC preconditioning can activate the expression levels of TRIF and IRF3 in the ischemic brain tissue and increase the generation of IFN-β [68,71], which further supports the protective effect of the TRIF-IRF signaling pathway in cerebral ischemia (Figure 1D). However, poly-IC preconditioning also significantly inhibited NF-κB activity and the generation of the pro-inflammatory cytokines TNF-α and IL-6 in the ischemic brain tissue and cultured cells [68,71]. Therefore, the inhibition effect of poly-IC preconditioning on the pro-inflammatory reaction cannot be excluded.

However, as discussed above, although TLR3 signaling is harmful to brain injury, it has no direct toxic effects in cerebral I/R injury. Thus, according to the preconditioning concept, the pretreatment of TLR3 ligand poly-IC, called TLR3 preconditioning, still elicits controversy. Further research needs to be conducted to confirm this concept of TLR3 preconditioning.

#### TLR4 ligand preconditioning and cerebral ischemic injury

The transient ischemic preexposure of brain tissue can lead to a tolerance of the brain tissue for the subsequently severe cerebral ischemia [72]. Clinically, the phenomenon of ischemic preconditioning tolerance has also been observed, and the transient ischemic attack could reduce the severity of the secondary ipsilateral stroke. Interestingly, ischemic preconditioning induced by 6 min of blockade in the bilateral carotid artery had a protective effect on the cerebral ischemia of wild-type mice; however, this protective effect disappeared in *TLR4*^*-/-*^ mice [73]. For the ischemic-preconditioning mice, the levels of TNF-α, iNOS, and COX-2 in the brain tissue were decreased, and this change was associated with TLR4-knockout mice [73]. These results show that TLR4 preconditioning is involved in the protective effect of ischemic preconditioning in the brain.

It has been reported that the activation of TLR4 by a small dose of LPS preconditioning could significantly reduce the severity of the damage in subsequent ischemic stroke. First, it was found that ischemic tolerance was induced in spontaneously hypertensive rats (SHR) by the injection of a single dose of LPS (0.9 mg/kg, i.v.) 1 to 7 days prior to the permanent MCAO [74]. Subsequently, it was found that in the mice treated with LPS (0.2 mg/kg) 48 h before ischemia induced by transient MCAO, LPS preconditioning induced significant neuroprotection against MCAO [75]. In a neonatal pig model of brain injury induced by cardiac arrest in hypothermia, LPS preconditioning induced robust protection against brain injury resulting from deep hypothermic circulatory arrest [76]. Recent studies have found that LPS preconditioning can also reduce brain damage in a neonatal HI rat model [77]. These results indicate that LPS preconditioning can induce a strong protective effect of cerebral ischemia for both permanent and transient cerebral ischemia models. Additionally, LPS preconditioning alleviates cerebral ischemic damage in adult rats, neonatal rats, mice, and neonatal piglets. Therefore, under the premise of a suitable dosage, LPS preconditioning provides a promising opportunity to prevent cerebral ischemic damage.

However, the exact molecular mechanism of ischemic tolerance induced by TLR4 activation with a non-lethal dose of LPS is unclear. It has been reported that TNF-α plays an important role in the ischemic tolerance in mice induced by LPS preconditioning [78]. LPS preconditioning significantly increased the level of TNF-α in blood prior to stroke, and TNF-α-deficient mice could not generate a protective effect of cerebral ischemia induced by LPS preconditioning. Additionally, the beneficial effect of LPS preconditioning in SHR was completely nullified by concurrent administration of TNFbp. The above results suggest that the tolerance to ischemia induced by LPS is likely to be mediated by TNF-α [78].

The inhibition of excessive inflammation of cerebral ischemia is a distinctive feature of LPS preconditioning. LPS preconditioning inhibits the neutrophil infiltration of the brain tissue and the activation of microglia surrounding the ischemic brain tissue, which are simultaneous with the inhibition of the mononuclear cells in peripheral blood [75]. Another study showed that the LPS stimulation in MCPIP1-deficient mice significantly increased the volume of cerebral infarction, accompanied by a significant upregulation of the pro-inflammatory cytokines in ischemic brain tissue, indicating that the protective effect of LPS preconditioning against cerebral ischemia was generated through the reduced level of pro-inflammatory cytokines by MCPIP1 [79]. Additionally, in the neonatal rat model of cerebral ischemia and hypoxia, LPS preconditioning upregulated the expression of eNOS in neurons and vascular endothelial cells by activating Akt, resulting in a protective effect against cerebral ischemia [77].

Genomic promoter analysis showed that 24 h after stroke, the expression levels of type I interferon in brain tissue were significantly increased in the mice with LPS preconditioning, as confirmed by the PCR assays [63]. TRIF activation after stroke can reduce the death rate of cortical neurons after OGD. In the focal MCAO model, the protective effect of LPS preconditioning against cerebral ischemia was dependent on the TLR4 signal transduction via TRIF. Additionally, IRF3-deficient mice could not produce a protective effect against cerebral ischemia [60,63]. These results indicate that IRF3 plays an important role in the protective effect of TLR4 activation by LPS preconditioning against cerebral ischemia, and LPS preconditioning alters the TLR signaling pathway after stroke and has a protective effect against cerebral ischemia mainly through the TRIF/IRF3 signaling pathway (Figure 1B).

#### TLR7 ligand preconditioning and cerebral ischemic injury

A recent study has shown that gardiquimod (GDQ) preconditioning of TLR7 ligand can also have a strong protective effect on the subsequent cerebral ischemia. At 72 h before MCAO, the subcutaneous injection of TLR7 ligand GDQ to mice significantly reduced the area of infarction and improved the neurological impairment [64]. In GDQ-pretreated mice, the transcription levels of five IFN-related genes, including Usp18, Oasl2, Isg15, and Ifit1, were significantly increased, indicating that GDQ preconditioning could change the gene response to stroke in mice, and the signaling of type I interferon plays an important role in the protection of cerebral ischemia by GDQ preconditioning. Additionally, compared with type I IFN (IFNAR)^+/+^ mice, the protective effect of cerebral ischemia in IFNAR^-/-^ mice with GDQ preconditioning was significantly decreased [64], indicating that TLR7 mediates the protection of cerebral ischemia through its cognate receptor IFNAR. Interestingly, GDQ preconditioning provided robust neuroprotection in TNF^-/-^ mice, indicating that TNF-α was not essential for protection. However, TNF-α is necessary for the ischemic tolerance induced by LPS and cytosine-phosphate-guanine (CpG) preconditioning (Figure 1E).

#### TLR8 ligand preconditioning and cerebral ischemic injury

Recently, it has been reported that the femoral venous injection of the TLR8 ligand R848 (1 mg/kg) 30 min prior to the MCAO in a mouse model could not produce the protective effect on cerebral ischemic damage in mice [58]. The reason may be related to the increasing infiltration of T cells and the apoptosis of neurons in the ipsilateral brain tissue after cerebral ischemia-reperfusion injury [58]; however, the exact cellular and molecular mechanism is not fully understood. Additionally, the effect of R848 preconditioning on the cerebral ischemia-reperfusion injury needs to be further verified, such as the optimization of the time point, the approach, and the R848 dosage.

#### TLR9 ligand preconditioning and cerebral ischemic injury

Studies have shown that TLR9 can cause ischemic tolerance [66]. The systematic injection of the TLR9 ligand CpG prior to cerebral ischemia reduced the cerebral ischemic damage by up to 60%, and the degree of this protection was dose dependent. Moreover, CpG preconditioning can produce the same protective effect on the cells *in vitro*. Additionally, the CpG-motif oligodeoxynucleotide (CpG-ODN) pretreatment has been shown to induce protection against cerebral I/R injury via the activation of PI3K/Akt signaling [80]. CpG preconditioning significantly increased the level of TNF-α in the serum of mice prior to MCAO, and TNF-α was essential to the subsequent protective effect of the brain because CpG preconditioning in TNF-α-knockout mice did not produce the protective effect against cerebral ischemia [66]. These results suggest that the protection against cerebral I/R injury by CpG preconditioning of TLR9 ligand and LPS preconditioning share a similar mechanism (Figure 1).

A recent study confirmed that the preconditioning of TLR4 and TLR9 ligands can induce similar genomic changes in brain tissue, mainly characterized by similar changes in the TLR signaling pathway. Both ligands can cause the transcription of IRF genes, thereby increasing the expression of type I interferon [81]. A phase II clinical trial of CpG-ODNs as an adjuvant drug and antitumor therapy is ongoing [82]. Therefore, CpG-ODNs may provide a good prospect for the prevention and treatment of cerebral ischemia.

### Mechanism of the protection against cerebral ischemia by TLR preconditioning

The preexposure of brain tissue to transient ischemia may cause tolerance to the subsequent severe ischemic damage. A recent study found that transient ischemia can induce subsequent changes in TLR signaling genes in the ischemic brain tissue, mainly in the 13 genes whose transcription is mediated by IRF. This change in the genetic profile was not found after a stroke without preconditioning [81]. These results suggest that TLR signals and IRFs play an important role in the cerebral tolerance induced by transient ischemia.

It had been found that the preexposure of LPS can induce a state of low reactivity in cells for subsequent secondary LPS stimulation, known as endotoxin tolerance. This state of low reactivity is related to the low expression of the LPS receptor complex TLR4/MD-2 on the cell surface, the reduced formation of TLR4-MyD88 complex, and the subsequent impairment of IRAK-1 activity [83]. The main characteristic of LPS-tolerant cells is that TLR4 stimulation cannot produce TNF-α. Unlike the primary cells, LPS-tolerant cells cannot recruit MyD88 to TLR4 and therefore do not activate IRAK-1 and NF-κB [83]. After the preexposure to LPS, with a precise negative feedback loop, the TLR4-NF-κB axis does not operate normally. For example, TRIM30 implemented the negative feedback regulation of the NF-κB activation mediated by TLRs through the degradation of TAB2 and TAB3 [84]. Additionally, enhancing Ship1 and Tollip inhibit TLR signaling, which leads to the suppression of NF-κB activity [60,85]. Additionally, the p50 precursor protein p105, which inhibits NF-κB activity by acting like an IκB molecule by sequestering NF-κB in the cytosol [60]. Thus, the subsequent TLR4-NF-κB signal was suppressed, and the production of pro-inflammatory cytokine was significantly reduced, thereby reducing the inflammatory response. The injection of a small dose of LPS in mice prior to MCAO was also able to generate tolerance to the subsequent cerebral ischemic damage, which was mainly dependent on the inhibition of TLR4 and the activation of the downstream signaling molecule NF-κB, thereby alleviating the inflammatory reaction of cerebral ischemia produced by this pathway [86]. When cerebral ischemic injury occurs, the endogenous ligands released from the damaged neurons might activate TLR4 and the downstream signaling pathways, resulting in the production of a large number of pro-inflammatory cytokines, and the activation of the inflammatory response may aggravate cerebral ischemic damage. However, the cellular pathways of TLR4 and cytokines could be altered in the mice with LPS preconditioning, which could suppress the immune response by increasing signal-inhibiting substances and decoying the receptors and anti-inflammatory cytokines. Thus, the induced immune suppression would reduce inflammation and the subsequent secondary cell death [60,86,87]. In contrast, the re-exposure of LPS increased the activity of the TLR4-IRF3 pathway, causing an increase in IFN-β production [88]. Additionally, the protection of cerebral ischemia induced by LPS preconditioning disappeared in TRIF- and IRF-knockout mice [60,81]. These results suggest that LPS preconditioning alters transcription in cells, inhibits the production of TNF-α and other pro-inflammatory factors induced by NF-κB, enhances the level of IFN-β induced by IRF3, alleviates the secondary inflammation after cerebral ischemia injury, and produces ischemic tolerance (Figure 1B).

Similar to the tolerance of LPS, the CpG preconditioning on TLR9 ligands caused a low reactive state for the subsequent CpG stimulation [89]. Moreover, TLR cross-tolerance can be generated between two TLRs because CpG preconditioning on TLR9 ligands can cause tolerance to the subsequent LPS stimulation of TLR4 [89,90]. LPS stimulation after CpG preconditioning on the cells would not only reduce TNF-α generation but also increase IFN-β generation [89]. After CpG preconditioning, the volume of cerebral infarction in mice was significantly reduced, and the level of TNF-α in peripheral blood was increased. However, the CpG preconditioning on TNF-α^-/-^ mice did not show a significant protective effect against cerebral ischemia, suggesting that TNF-α is necessary for CpG-induced ischemic tolerance [66]. These results indicate that LPS and CpG preconditioning share a common protection mechanism. Additionally, the direct CpG preconditioning for the culture of mixed cortical cells can produce a protective effect on the subsequent mixed cortical cells with OGD. CpG treatment may modulate the cytokine response to injury in glial cells, which in turn may have a protective effect on neurons. However, its mechanism has not yet been fully elucidated [66].

### TLR ligand postconditioning and cerebral ischemic injury

Recently, some studies have shown that the modification of reperfusion after cerebral ischemia can produce a protective effect against cerebral ischemia, and this phenomenon is known as postconditioning. For *in vivo* and *in vitro* models, postconditioning 10 min after the ischemic reperfusion for 10 min achieved the greatest protective effect, with the infarction area reduced by 50% and the cell death rate reduced by 30% [91]. The level of protection of ischemic postconditioning against cerebral ischemia is similar to that of the preconditioning; however, the mice that received both ischemic preconditioning and postconditioning did not exhibit a greater protective effect [91], indicating that the ischemic preconditioning and postconditioning may share a similar signaling mechanism. Recent studies have shown that ischemic postconditioning of TLRs could also produce a protective effect against cerebral ischemia by inducing the TLR ischemic tolerance [67,69,80,92] (Table 2). This result provides a new approach for the treatment of cerebral ischemia.

**Table 2:** **TLRs postconditionings and effects**

**Animal**	**Model**	**Postconditioning time/approach**	**TLR/ischemia**	**Results/mechanism**	**Author**	**References**
Mice	MCAO, ischemia 2 h	CCA 10-s ligation, subsequently 30-s open, a total of 3 cycles	Ischemic postconditioning	Decrease brain infarction volume and improve neurological function. Activate AKT signaling pathway	Gao X	[92]
Mice	MCAO, 60-min ischemia, 24-h reperfusion	30 min/systemic injection	TLR2, Pam3CSK4	Decrease brain infarction volume and improve neurological function. Activate TLR2/PI3K/Akt signaling pathway	Lu C	[65]
Mice	MCAO, 60-min ischemia, 24-h reperfusion	30 min/IP	TLR3 poly-IC	Decrease brain infarction and prevention of Fas/FADD interaction	Zhang X	[67]
Mice	MCAO, 60-min ischemia, 48-h reperfusion	3 h/IP	TLR3 poly-IC	Decrease brain infarction volume and improve neurological function. Downregulate TLR4 signaling pathway by TLR3	Wang PF	[90]
Mice	MCAO, 60-min ischemia, 24-h reperfusion	15 min/IP	TLR9 CpG-ODN	Decrease brain infarction and activation of PI3K/Akt Signaling	Lu C	[78]

MCAO: middle cerebral artery occlusion; IP: intraperitoneal injection; CCA: common carotid artery TLR: toll-like receptor; Poly-IC: polycytidylic acid; FADD: Fas-Associated protein with Death Domain; CpG-ODN: CpG-motif oligodeoxynucleotide. MCAO, 60-min ischemia, 24-h reperfusion: the mice was employed MCAO (cerebral ischemia) 60 min followed by reperfusion 24 h. 3 h/IP: Mice were injected intraperitoneally with TLRs ligand 3 h after cerebral ischemia.

#### TLR2, TLR9 ligand postconditioning and cerebral ischemic injury

Intraperitoneal injection of TLR2 ligand Pam3CSK4 30 min after cerebral ischemia significantly reduced the infarction area [67]. Pam3CSK4 postconditioning increased the levels of Hsp27, Hsp70, and Bcl2 in the ischemic brain tissue and reduced Bax levels and NF-κB activity. Importantly, compared with the control group, the phosphorylation levels of Akt/Akt and GSK-3b/GSK-3b in the Pam3CSK4 postconditioning group were significantly increased, suggesting that the postconditioning of TLR2 ligands alleviated the focal cerebral ischemic injury mainly through a TLR2/PI3K/Akt-dependent mechanism. It has also been demonstrated that the administration of TLR9 ligand CpG-ODN 15 min after cerebral ischemia significantly reduced cerebral I/R injury via a PI3K/Akt-dependent mechanism [80].

#### TLR3 ligand postconditioning and cerebral ischemic injury

**Figure 2:**
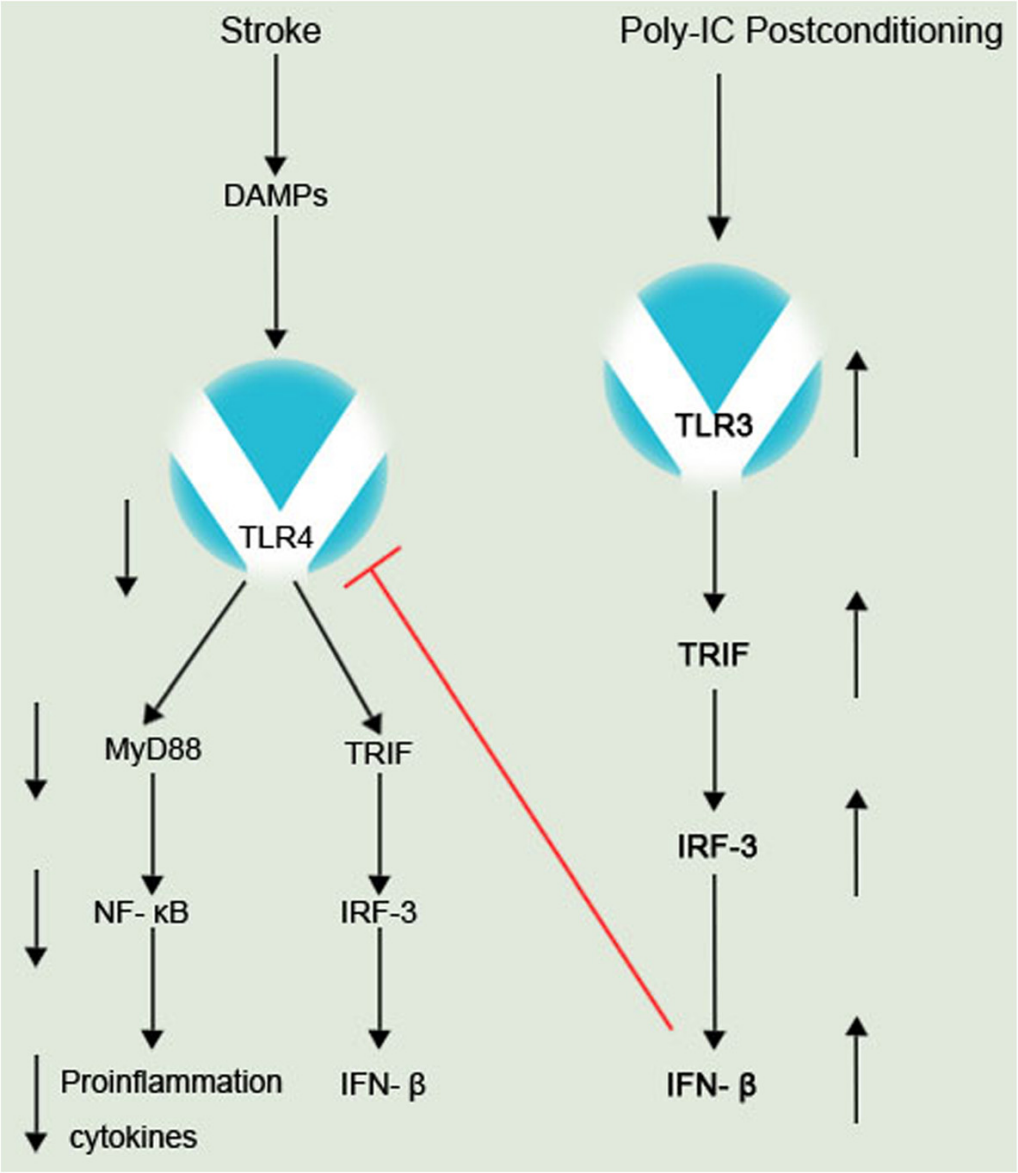
Poly-IC postconditioning activates the TLR3/IRF3 signaling pathway and increases the IFN-β levels and subsequently downregulates TLR/NF-κB signaling to decrease the levels of pro-inflammatory cytokines. DAMPs, damage-associated molecular patterns; TLR, toll-like receptor; TRIF, TIR-domain-containing adapter-inducing interferon-β; IRF, interferon regulatory factor; IFN, interferon; Poly-IC, polycytidylic acid.

For the first time, we found that the postconditioning with the TLR3 ligand poly-IC can reduce cerebral ischemic injury. The intraperitoneal injection of poly-IC 3 h after cerebral ischemia significantly reduced the volume of cerebral infarction and the brain water content and improved the neurological function score [92]. Poly-IC treatment reduced the levels of TNF-α and IL-1β in the ischemic brain tissue, cerebrospinal fluid, and peripheral blood and increased IFN-β levels, suggesting that poly-IC plays an important role in the protection of cerebral ischemia by reducing the inflammatory processes. However, in TLR3-knockout mice, the protective effect of poly-IC against cerebral ischemia disappeared. Meanwhile, our findings showed that poly-IC downregulated the TLR4 signaling pathway and NF-κB activity in ischemic brain tissue, suggesting that the cerebral protective effect of poly-IC is achieved by the downregulation of the TLR4 signaling pathway through TLR3. Importantly, poly-IC showed a long-term protective effect against cerebral I/R injury as well as a protective effect against permanent cerebral ischemic injury in rats [92] (Figure 2). Other results showed that the therapeutic administration of poly-IC administered 30 min after cerebral ischemia markedly decreased infarct volume via TLR3-mediated prevention of Fas/FADD interaction [69], suggesting that poly-IC postconditioning plays a protective role against cerebral I/R injuries. However, as with the concept of TLR3 preconditioning, the concept of TLR3 postconditioning still needs to be verified.

### Mechanisms for the protection of TLR postconditioning against cerebral ischemia

The mechanism of cerebral ischemia protection by postconditioning is not yet fully understood. Currently, it is generally believed that the main target of treatment after ischemia is early reperfusion to reduce the generation of free radicals and inflammation, reduce brain edema and blood-brain barrier leakage, and inhibit apoptosis [93]. Moreover, the postconditioning of ischemia increased Akt phosphorylation, and the inhibition of Akt partially weakened the protective effect of the postconditioning of ischemia [94], suggesting that the Akt signaling pathway plays an important role in the protective effect of the postconditioning. Additionally, the extracellular signal regulated kinase (ERK) for pro-survival enzyme and the mitogen-activated protein kinase (MAPK) also participated in the protective effect against ischemic brain injury [91].

The protective mechanism of TLR postconditioning against cerebral ischemia requires further investigation, and the mechanism of TLR postconditioning is partially similar to that of the postconditioning of ischemia. For example, postconditioning with the TLR2 ligand Pam3CSK4 and TLR9 ligand CpG-ODN induced protection against cerebral ischemia primarily through the PI3k/Akt signaling pathway [67,80]. TLR2 can form a dimer with the PI3 enzyme to increase the activity of the P-Akt signaling pathway and promote the anti-apoptotic effect of the cerebral ischemia protective agent, resulting in the cerebral ischemia protection [67], indicating that the PI3-Akt signaling pathway plays an important role in the brain protection of TLR2 ligand Pam3CSK4 and TLR9 ligand CpG-ODN postconditioning.

Our study showed that poly-IC postconditioning with the TLR3 ligand could reduce the expression of ischemic brain tissue and microglial cells, inhibit the TLR4/MyD88 signaling pathway, reduce NF-κB activity, and reduce the production of inflammatory cytokines, such as TNF-α and IL-β. Additionally, the downregulation of the TLR4 signaling pathway is mediated by TLR3 [92] (Figure 2). Additionally, the decreased cerebral I/R injury by poly-IC via TLR3 was associated with the prevention of the interaction of Fas and FADD, as well as microglial cell caspase-3 and caspase-8 activities [69].

### The clinical significance of TLR ischemic tolerance in cerebral ischemia

In clinical practice, approximately 50% of the patients with coronary artery bypass surgery suffer a long-term decline in cognitive function due to the surgical complications of local or global cerebral ischemia [95]. In carotid endarterectomy and carotid artery stent implantation, ischemic brain damage is a severe complication; therefore, for these high-risk patients, a prophylactic treatment for cerebral protection is very beneficial. Although ischemic preconditioning can reduce the subsequent cerebral ischemic damage, it is difficult for high-risk patients to tolerate; therefore, the TLR preconditioning for the protective effect against cerebral ischemia may increasingly attract attention. As mentioned previously, the preconditioning of several TLR ligands significantly reduced cerebral ischemia-reperfusion injury in mouse and rat models of cerebral ischemia. Importantly, one study showed that the drug preconditioning of the TLR9 ligand CpG-ODNs could also produce a protective effect in a model of cerebral ischemic injury in the non-human primate rhesus monkey, and the reagents applied in this experiment have been used in human clinical trials, which provides a new preventive approach for patients at high risk of cerebral ischemia [96]. Moreover, the TLR3 ligand poly-IC has been used as an adjuvant therapy and antitumor drug in clinical applications [97,98], and its safety for the human body has been confirmed, suggesting that poly-IC is a promising precursor of the prevention drug for patients at high risk for cerebral ischemia.

Although the preconditioning may be beneficial to certain patients at risk of cerebral ischemia, patients with acute stroke need an effective treatment after onset; therefore, in recent years, increasing attention has been focused on the postconditioning after the onset of stroke. Postconditioning of ischemia can reduce the cerebral edema and the leakage of the blood-brain barrier, as well as block apoptosis by reducing the generation of free radicals and inflammation, thereby reducing cerebral ischemic damage. The postconditioning of cardiac ischemia has been applied in clinical trials [99]. Therefore, the cerebral ischemic tolerance induced by TLR postconditioning provides a broad prospect of clinical application. Ours and others’ study results showed that poly-IC postconditioning reduced cerebral ischemic-reperfusion injury [69,92]; therefore, poly-IC is a promising drug precursor for the treatment of stroke. However, the clinical trial of the TLR postconditioning for ischemic stroke is still facing some unsolved issues, such as the choice of the stroke patients and the effective time point for the TLR3 postconditioning. However, although the issues, including whether TLRs postconditioning can extend the thrombolytic time window of r-tPA and what the long-term effects are, urgently need to be solved, this technique still provides a good possibility for treating ischemic stroke.

## Conclusions and prospects

Cerebral ischemic injury occurs due to a series of complex pathophysiological events. It has been clear that the inflammation following ischemia mediated by TLRs plays an important role in secondary brain injury. Interestingly, the activation of TLR signals before cerebral ischemia can reduce the subsequent ischemic injury caused by severe stroke, which is defined as preconditioning or TLR ischemic tolerance. To date, cerebral ischemic damage can be reduced after the tolerance of several TLRs was induced individually. However, the protection mechanism of the TLR preconditioning has not yet been fully elucidated. Currently, it is believed that TLR preconditioning can alter TLR signaling pathways after ischemia, mainly manifested as the activation of the TLR4/TRIF signaling pathway and the inhibition of the TLR4/MyD88 signaling pathway, thereby increasing the production of anti-inflammatory cytokines, reducing the generation of pro-inflammatory cytokines [81], and reducing the inflammatory damage of cerebral ischemia.

In recent years, postconditioning of ischemia has also attracted attention. Our study and other studies have shown that postconditioning with TLR2, TLR3, and TLR9 ligands could significantly reduce cerebral ischemic injury [67,80,92], which broadens the role of TLRs in cerebral ischemia and provides a direction for the further research in the protective mechanism of endogenous cerebral ischemia induced by TLR postconditioning. Therefore, TLR postconditioning also provides a new treatment approach for ischemic stroke. Moreover, the TLR3 ligand poly-IC and the TLR9 ligand CpG ODNs have been applied for other clinical indications, showing significant efficacy and safety. Therefore, treatments targeting TLRs may represent a good prevention strategy for patients at a high risk of stroke.

## Abbreviations

DAMPs: damage-associated molecular patterns
I/R: ischemic reperfusion
IRAK: IL-1R-associated kinase
LPS: lipopolysaccharide
MCAO: middle cerebral artery occlusion
NPCs: nerve primitive cells
ODG: oxygen-glucose deprivation
PAMPs: pathogen-associated molecular patterns
Poly-IC: polycytidylic acid
PRRs: pattern recognition receptors
RGC: retinal ganglion cell
TAB1: TAK1-binding protein 1
TAK1: TGF-b-activated kinase 1
TBK1: TANK-binding kinase 1
TLRs: toll-like receptors
